# Roflumilast Inhibits Respiratory Syncytial Virus Infection in Human Differentiated Bronchial Epithelial Cells

**DOI:** 10.1371/journal.pone.0069670

**Published:** 2013-07-23

**Authors:** Manuel Mata, Isidoro Martinez, Jose A. Melero, Herman Tenor, Julio Cortijo

**Affiliations:** 1 Research Foundation of the University General Hospital of Valencia, Valencia, Spain; 2 Centro de Investigación Biomédica en Red (CIBER) de Enfermedades Respiratorias, Valencia, Spain; 3 University of Valencia, Valencia, Spain; 4 Unidad de Interacción Virus-Célula, Centro Nacional de Microbiología, Instituto de Salud Carlos III, Madrid, Spain; 5 Unidad de Biología Viral, Centro Nacional de Microbiología, Instituto de Salud Carlos III, Madrid, Spain; 6 Centro de Investigación Biomédica en Red (CIBER) de Enfermedades Respiratorias, Instituto de Salud Carlos III, Madrid, Spain; 7 Takeda Pharmaceuticals International, Zürich, Switzerland; University of Iowa, United States of America

## Abstract

Respiratory syncytial virus (RSV) causes acute exacerbations in COPD and asthma. RSV infects bronchial epithelial cells (HBE) that trigger RSV associated lung pathology. This study explores whether the phosphodiesterase 4 (PDE4) inhibitor Roflumilast N-oxide (RNO), alters RSV infection of well-differentiated HBE (WD-HBE) *in vitro*. WD-HBE were RSV infected in the presence or absence of RNO (0.1-100 nM). Viral infection (staining of F and G proteins, nucleoprotein RNA level), mRNA of ICAM-1, ciliated cell markers (digital high speed videomicroscopy, β-tubulin immunofluorescence, Foxj1 and Dnai2 mRNA), Goblet cells (PAS), mRNA of MUC5AC and CLCA1, mRNA and protein level of IL-13, IL-6, IL-8, TNFα, formation of H_2_O_2_ and the anti-oxidative armamentarium (mRNA of Nrf2, HO-1, GPx; total antioxidant capacity (TAC) were measured at day 10 or 15 post infection. RNO inhibited RSV infection of WD-HBE, prevented the loss of ciliated cells and markers, reduced the increase of MUC5AC and CLCA1 and inhibited the increase of IL-13, IL-6, IL-8, TNFα and ICAM-1. Additionally RNO reversed the reduction of Nrf2, HO-1 and GPx mRNA levels and consequently restored the TAC and reduced the H_2_O_2_ formation. RNO inhibits RSV infection of WD-HBE cultures and mitigates the cytopathological changes associated to this virus.

## Introduction

Respiratory syncytial virus (RSV) is an enveloped negative-sense single-stranded RNA virus of the *Paramyxoviridae* family. It is the main cause of severe lower respiratory tract infections (bronchiolitis and pneumonia) in infants and young children and it is also a pathogen of considerable importance in the elderly and in immunocompromised adults [1,2]. In addition, a substantial proportion of acute exacerbations in COPD or asthma have been related to RSV infections [3,4]. In COPD, acute exacerbations attributed to RSV may account for about 10.000 deaths per year among the elderly population (> 65 years of age) in the US [5].

The main target of RSV is the ciliated bronchial epithelial cells. RSV infection triggers a loss of ciliated cells and Goblet cell metaplasia associated with enhanced MUC5AC production [6–9]. Furthermore, RSV infection causes the release of numerous pro-inflammatory chemokines and cytokines from bronchial epithelial cells and entails an imbalance between an enhanced reactive oxygen species (ROS) production and a compromised antioxidant enzymatic armamentarium [10,11]. Collectively, given these numerous effects, bronchial epithelial cells are considered key players of RSV-induced lung diseases.

Among the new concepts in respiratory diseases, such as COPD and asthma, selective inhibitors of phosphodiesterase 4 (PDE4) are under scrutiny for more than two decades as new selective drugs. PDE4 is one out of the eleven families of cyclic nucleotide hydrolyzing phosphodiesterases in humans and uses cAMP as its specific substrate. As a corollary, inhibitors of PDE4 augment cellular cAMP content resulting in inhibitory effects on inflammation, oxidative stress and tissue remodeling [12–14]. PDE4 is expressed in human airway epithelial cells [15–17]. While initial studies largely failed to demonstrate significant effects of PDE4 inhibitors on airway epithelial cells [16,17], evidence is accumulating from more recent reports that selective inhibitors of PDE4 can: i) reduce EGF-stimulated MUC5AC expression [18], ii) enhance CFTR activity [19], iii) prevent TGFβ1-induced epithelial mesenchymal transition [20], iv) promote ciliary beat frequency and protect from tobacco smoke-induced loss of ciliated cells [21] and v) reduce the release of a number of cytokines or chemokines following different stimuli of primary human bronchial epithelial cells or established cell lines such as A549 or BEAS2B [22–25].

Historically the development of PDE4 inhibitors was intimately related to COPD and as the first-in-class PDE4 inhibitor roflumilast is currently in use for the treatment of severe COPD in patients with chronic bronchitis and frequent exacerbations. A major asset inherent to this remedy is its proven ability to mitigate the risk of acute exacerbations [26–30], which might be triggered by viral infections.

Evidence has been provided that a range of therapeutic compounds of potential interest in COPD such as statins [31], anti-oxidants such as N-acetylcysteine or L-carbocisteine [9,32], tiotropium [33], macrolides [34] and PPARγ agonists [35] are all capable to reduce RSV production in human bronchial epithelial cells.

However, effects of PDE4 inhibitors and specifically roflumilast on RSV-infected bronchial epithelial cells have not yet been explored. Therefore, the objective of the current study was to analyze the effects of the PDE4 inhibitor roflumilast-N-oxide (RNO, the active metabolite of roflumilast largely governing clinical efficacy [36,37]) in an *in vitro* model of RSV infection in well-differentiated normal human bronchial epithelial cells (WD-HBE). In this context it was explored whether the PDE4 inhibitor influences viral load, ICAM-1 expression, markers of ciliated cells (β-tubulin, Foxj1, Dnai2) and Goblet cells (MUC5AC and CLCA1), a range of inflammatory cytokines (IL-13, IL-6, IL-8, TNFα) and the burden of oxidative stress and the anti-oxidative cellular armamentarium.

## Materials and Methods

### Cells, infections and incubations

Human lung tissue was obtained from patients subjected to surgery for lung carcinoma as previously described [18]. Procedures were approved by the local ethics committee. Te full name of this Commit is: "Comite Etico de Investigacion Clinica del Consorcio

Hospital General Universitario de Valencia". Written informed consent of all donors were obtained. At the time of surgery, lung function was within the normal range (spirometry). WD-HBE cells were cultured and differentiated in 24 wells transwell inserts (0.3 cm^2^, Corning Costar, High Wycombe, UK) under air-liquid interface (ALI) conditions as previously described [9]. WD-HBE cells were infected with 2 × 10^6^ plaque forming units (PFU) of RSV or mock in 100 µL of differentiation medium per insert in the presence or absence of RNO 0.1-100 nM. Cultures were incubated for 2 hours at 37^°^C and washed once with 500 µL of differentiation medium. RNO was added to the cultures for 1h prior to infections and remained present until the end of the experiment.

Cultures were maintained until day 15 post-infection and culture medium and analysed compounds were replaced every other day. Measurements were performed at the indicated time points over this period. In this study, cultures from three different donors were used.

RNO was obtained from Nycomed GmbH: A Takeda Company (Konstanz, Germany). It was diluted from a 10mM stock in DMSO to the final concentrations in 0.1% DMSO.

### RSV production and determination of viral titters

RSV (Long Strain, ATCC VR-26) obtained from the ATCC (American Type Culture Collection; Rockville, MD, USA) was propagated in HEp-2 cells as previously described [38]. Viruses were purified from clarified culture supernatants by polyethylene glycol precipitation and centrifugation in a 30–45–60% discontinuous sucrose gradient in TNE buffer [39].

Viral titters were determined by plaque assay in HEp-2 cells with 0.5% low melting-point agarose overlaid (Conda Laboratories, Madrid, Spain). Virus inactivation (for mock-infection) was achieved by irradiation with UV light for 90 min and confirmed by plaque assay.

### Quantification of viral infection

Viral infection in WD-HBE cultures was determined by immunocytochemistry of viral glycoproteins G and F and real-time RT-PCR as previously described [9].

Immunocytochemistry of viral glycoproteins was conducted with a pool of monoclonal antibodies (2F, 47F, 56F, 021/1G, 021/2G). For semi-quantitative Real Time RT-PCR, primers to amplify the nucleoprotein RNA of the human RSV (forward primer 5´ CATGATTCTCCTGATTGTGGGATGA3´, reverse primer: 5´ TCACGGCTGTAAGACCAGATCTAT 3´, probe: 5´ CCCCTGCTGCCAATTT3´) were designed [9]. The procedures for RNA extraction, cDNA synthesis and real time PCR were as described later. Ct values obtained for the RSV nucleoprotein RNA were related to GAPDH of the bronchial epithelial host cells and finally presented according to the standard 2-ΔΔCt procedure.

### Functional ciliated cell analysis

Ciliary beat frequency and motility were evaluated as described previously by digital high speed videomicroscopy (DHSV) [9,40]. Three independent videos of each insert were recorded and the number of cells with cilia motility was assessed by manual counting by a blinded observer.

### Immunofluorescence analysis of β-tubulin

Nitrocellulose membranes were removed from the inserts, placed in Tissue-Tek® OCT^TM^ (Sakura Finetek, Europe, Alphen aan den Rijn, The Netherlands) and cut into 10 µm sections. Immunofluorescence was performed using a mouse monoclonal antibody against human β-tubulin and a secondary rhodamine labeled goat anti-mouse IgG (Sigma, St Louis, MO, USA). DAPI (Invitrogen Ltd., Paisley, UK) was used for nuclear staining. Ten sections of each group were analyzed and the results were shown as percent of mock-infected as previously described [41].

### Analysis of Goblet cells by PAS staining

PAS staining served to identify Goblet cells in WD-HBE. Nitrocellulose membranes were removed from the inserts, fixed and paraffin-embedded as outlined before [18]. Inserts were cut in 5µm sections and stained using the PAS staining system (Sigma, St Louis, MO, USA). Finally, sections were mounted with DPX (Sigma, St Louis, MO, USA) prior to microscopical analysis.

### Gene expression and protein release analyses

Expression of MUC5AC, ICAM-1, hCLCA1, TNFα, IL-8, IL-6, IL-13, Nrf2, HO-1 and GPx was quantified by real-time RT-PCR as described previously [18] using designed primers/probes (Assays-on-Demand, Applied Biosystems, Foster City, CA, USA). References of assays on demand used in this study are Hs00873651_m (MUC5AC), Hs00164932_m1 (ICAM-1), Hs00976287_m1 (hCLCA1), Hs01113624_g1 (TNFα), Hs00174103_m1 (IL8), Hs00985639_m1 (IL6), Hs00174379_m1 (IL13), Hs00975961_g1 (Nrf2), Hs01110250_m1 (HO-1) and Hs00829989_g (GPX). The 2^-ΔΔCt^ method was used to obtain semi-comparative data.

WD-HBE were washed once with 100µL of sterile PBS, and 65 µL of fresh culture medium was added to the surface. After one hour of incubation, supernatants were collected and analyzed for the release of TNF-α, IL-8 and IL-6 using a multiplex cytometer-based method (Luminex DX100, Luminex Corp., Austin TX, USA) and ultrasensitive panels (Millipore, Billerica, MA, USA). Determinations were carried out following manufacturer instructions.

### Determination of Total Antioxidant Capacity (TAC) and H_2_O_2_ concentration

TAC was determined in whole cell lysates using the Total Antioxidant Assay kit (Cayman Chemical Company, Michigan, USA) following manufacturer’s instructions. Intracellular levels of H_2_O_2_ were determined using the Amplex Red Reagent (Invitrogen Ltd., Paisley, UK) as previously described [42].

### Analysis of results

Data are shown as the mean ± SEM. Statistical analysis was performed by analysis of variance (ANOVA) followed by Tukey’s multiple comparison test or t-test, as appropriate (GraphPad Software Inc., San Diego, CA). Significance was accepted at p<0.05. EC50 values were calculated from non-linear regression analyses using GraphPad Prism Software.

## Results

### Effects of roflumilast *N-*oxide on RSV replication and density of cells with cilia motility in WD-HBE

**Figure 1:**
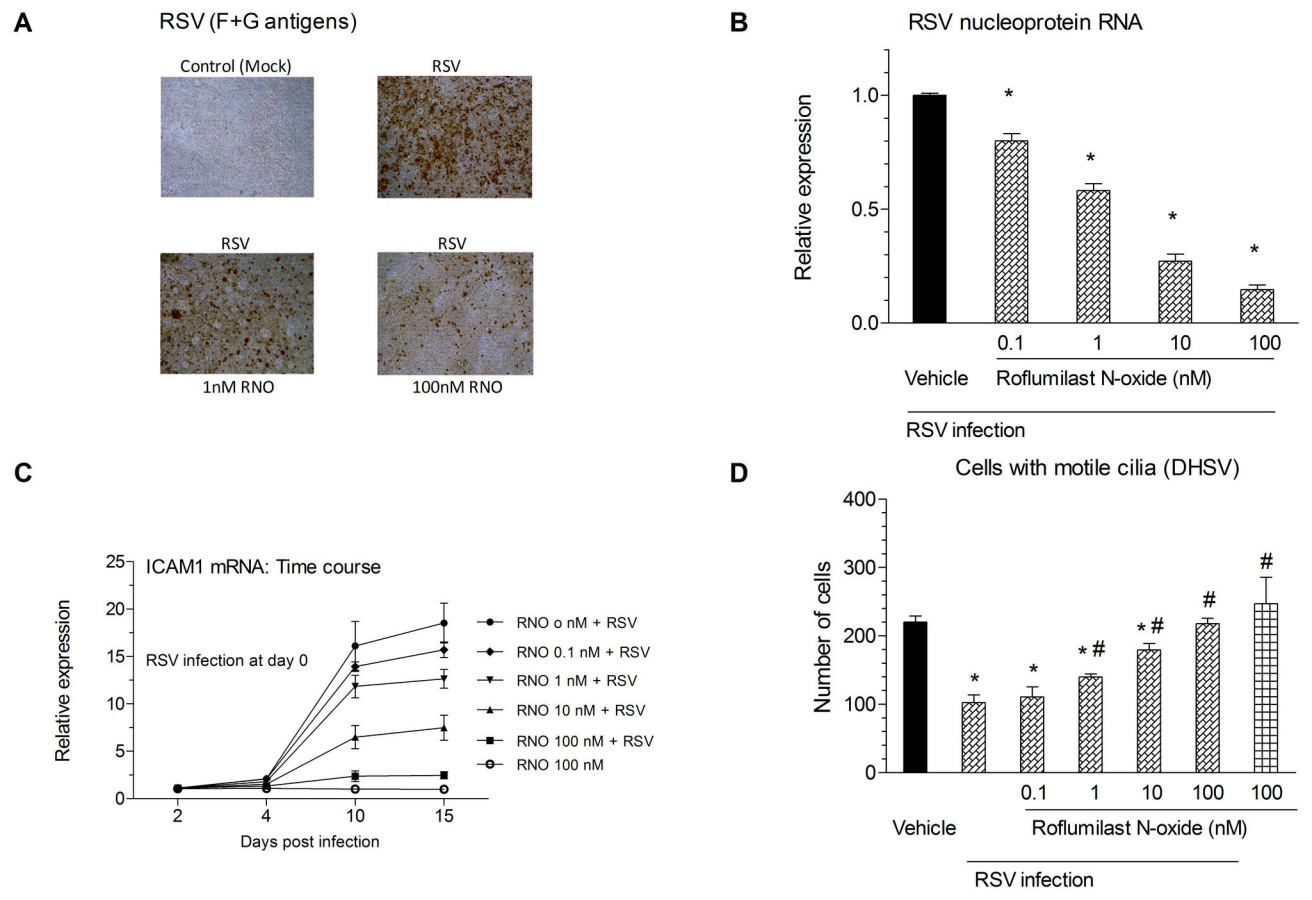
Roflumilast N-oxide (RNO) alleviated viral burden following RSV infection, reduced RSV-induced ICAM-1 expression and restored cilia motility in well-differentiated human bronchial epithelial cells (WD-HBE). WD-HBE were infected with RSV (2 x 10^6^ PFU/insert) or mock-infected (control) in the presence of Roflumilast N-oxide (RNO; 0.1-100nM) or vehicle and maintained in air-liquid culture over another 10 days for viral quantification or 15 days for ICAM-1 and cilia motility evaluation. RSV abundance in WD-HBE was determined by immunocytochemical detection of RSV F and G proteins (panel A) or real-time RT-PCR based quantification of RSV nucleoprotein RNA (panel B). In panel B viral nucleoprotein RNA was related to host cell GAPDH and normalized to RSV infection with vehicle (=1). In mock-infected WD-HBE, RSV nucleoprotein RNA was below detection limit. ICAM1 expression was determined by Real Time RT-PCR using mock-infected cells as controls (panel C). Cilia motility was evaluated by HSDV using three independent videos of each experimental replicate. Results represent mean ± SEM of three independent infections. Each condition was evaluated by triplicate in three independent wells. Cultures from three different patients were used. * p< 0.05 compared to mock-infected cells. # p<0.05 compared to infected cells.

First, the effects of RNO on the viral load in RSV-infected well-differentiated normal human bronchial epithelial cells (WD-HBE) were investigated. WD-HBE were infected with RSV (2 x 10^6^ PFU/insert, 2 h) in the absence or presence of RNO (0.1-100 nM) or mock-infected. At day 10 after RSV infection there was a very significant increase in intracellular viral antigens as visualized by immunocytochemical detection of viral F and G proteins (Figure 1A, upper right versus left microphotograph). RNO reduced the number of RSV antigen positive cells in a concentration-dependent manner (Figure 1A, lower panels). In parallel, WD-HBE infected with RSV showed a substantial increase in the expression of RSV nucleoprotein RNA that was reduced steadily by increasing doses of RNO (Figure 1 panel B) in a concentration-dependent manner (EC50 of 1.3 nM).

Next, RNA levels of the intercellular adhesion molecule 1 (ICAM1) were measured because this molecule facilitates RSV entry and infection of human epithelial cells [Behera et al., 2001]. ICAM-1 mRNA expression was increased to about 16-fold and 18-fold at day 10 and 15 post RSV infection. RNO inhibited this induction in a concentration-dependent manner (EC50 of 5.3 nM, Figure 1 panel C).

Next, the number of cells with motile cilia was evaluated. WD-HBE were infected with RSV in the presence of RNO (0.1-100nM) or vehicle. At day 10 after RSV infection, the number of cells with motile cilia decreased by about 55% compared to mock-infected cells. RNO prevented this reduction of ciliated cells in a concentration-dependent manner (EC50 of 5.9 nM) being significant at 1nM and fully prevented the effect of RSV at 100nM (Figure 1 panel D)

### Effects of RSV and RNO on the number of β-tubulin labelled ciliated cells and mRNA levels of the ciliated cell markers foxj1 and Dnai2

**Figure 2:**
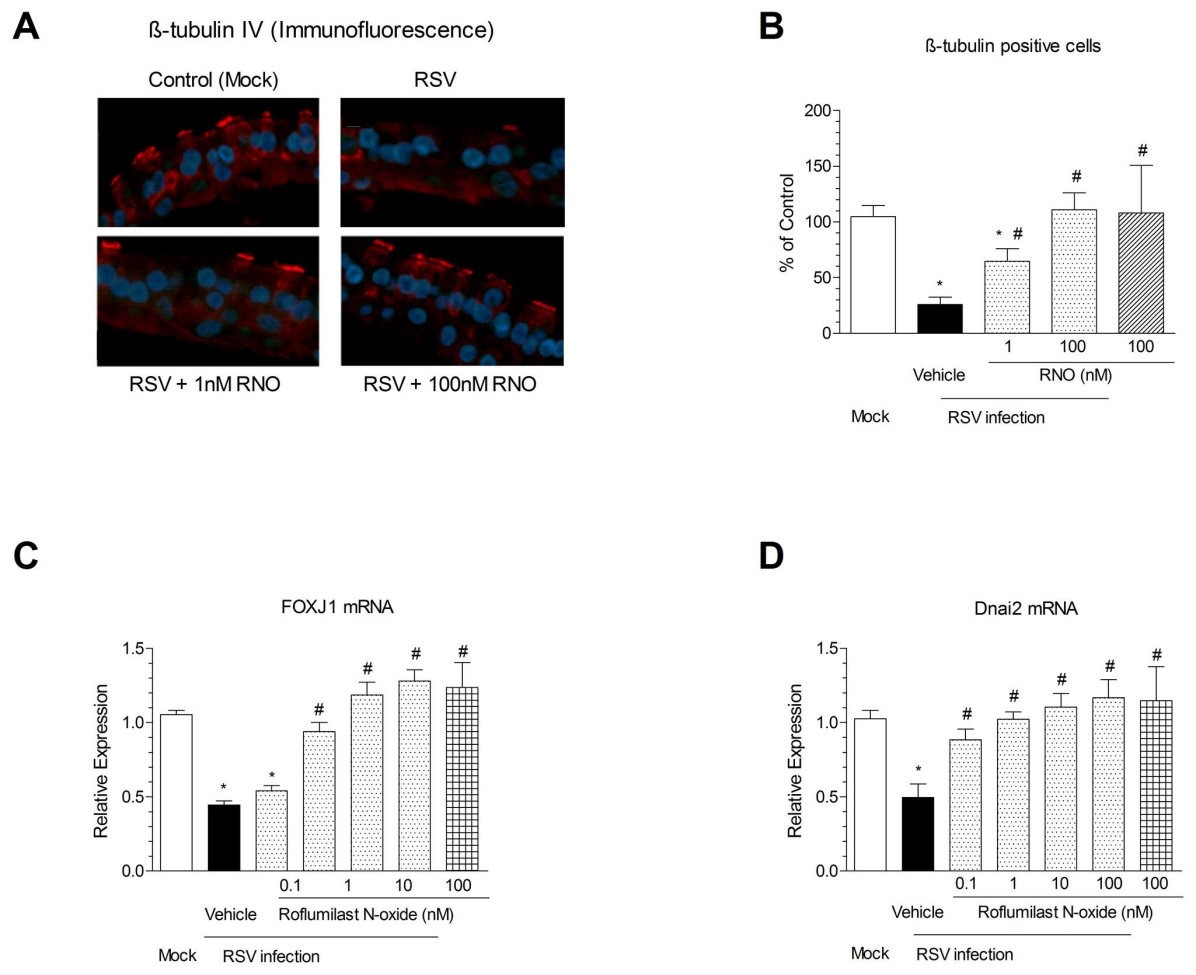
RSV compromised the number of β-tubulin IV labeled ciliated cells and the expression of Foxj1 and Dnai2: Reversal by roflumilast N-oxide. WD-HBE were infected with RSV at 2 x 10^6^ PFU/insert or mock infected and cultured in the presence of roflumilast N-oxide (RNO; 0.1-100nM) or vehicle (0.1% DMSO) over 10 days. To evaluate the number of ciliated cells sections from WD-HBE cells were immunostained for β tubulin IV as described in Methods. In (A) representative microphotographs from mock infected, RSV infected with vehicle, RSV infected with 1nM roflumilast N-oxide, RSV infected with 100nM roflumilast N-oxide sections are shown. β-tubulin IV staining is red (rhodamine), nuclei are blue (DAPI). The microphotographs are representative from three separate infections. For quantitative analyses the numbers of β-tubulin IV staining (ciliated) cells were counted in ten different sections of three separate infections and expressed as percent of ciliated cells in mock-infected, untreated controls (=100%) (panel B). At day 10 after infection total RNA of WD-HBE was extracted and the expression of Foxj1 (panel C) and Dnai2 (panel D) was quantified by real time RT-PCR in mock-infected (white bars) or RSV infected cells in the presence of roflumilast N-oxide (RNO; 0.1-100 nM; grey bars) or vehicle (black bars). Results are depicted as the means ± SEM of three independent infections. Each condition was evaluated by triplicate in three independent wells. Cultures from three different patients were used. * p< 0.05 compared to mock-infected cells. #p<0.05 versus RSV infected cells.

Next it was addressed whether roflumilast N-oxide may reverse a loss in cilia following RSV [9] that may account for the effects on the number of cells with motile cilia. To this end, WD-HBE were infected with RSV (2 × 10^6^ PFU/insert) in the absence or presence of RNO at 1 or 100nM, or mock infected. At day 10 after infection sections were immunostained to detect β tubulin IV. RSV reduced by 75% the apical β tubulin IV staining. RNO concentration-dependently rescued this reduction by 50% (Figure 2 panels A and B). This cilia loss was consistent with the results obtained for the gene expression of the ciliagenesis regulator FOXJ1 and the dynein DNAI2 (Figure 2 panels C and D respectively).

### Effect of roflumilast N-oxide on RSV-induced Goblet cell metaplasia and the enhanced expression of MUC5AC and hCLCA1, potential role of IL-13

**Figure 3:**
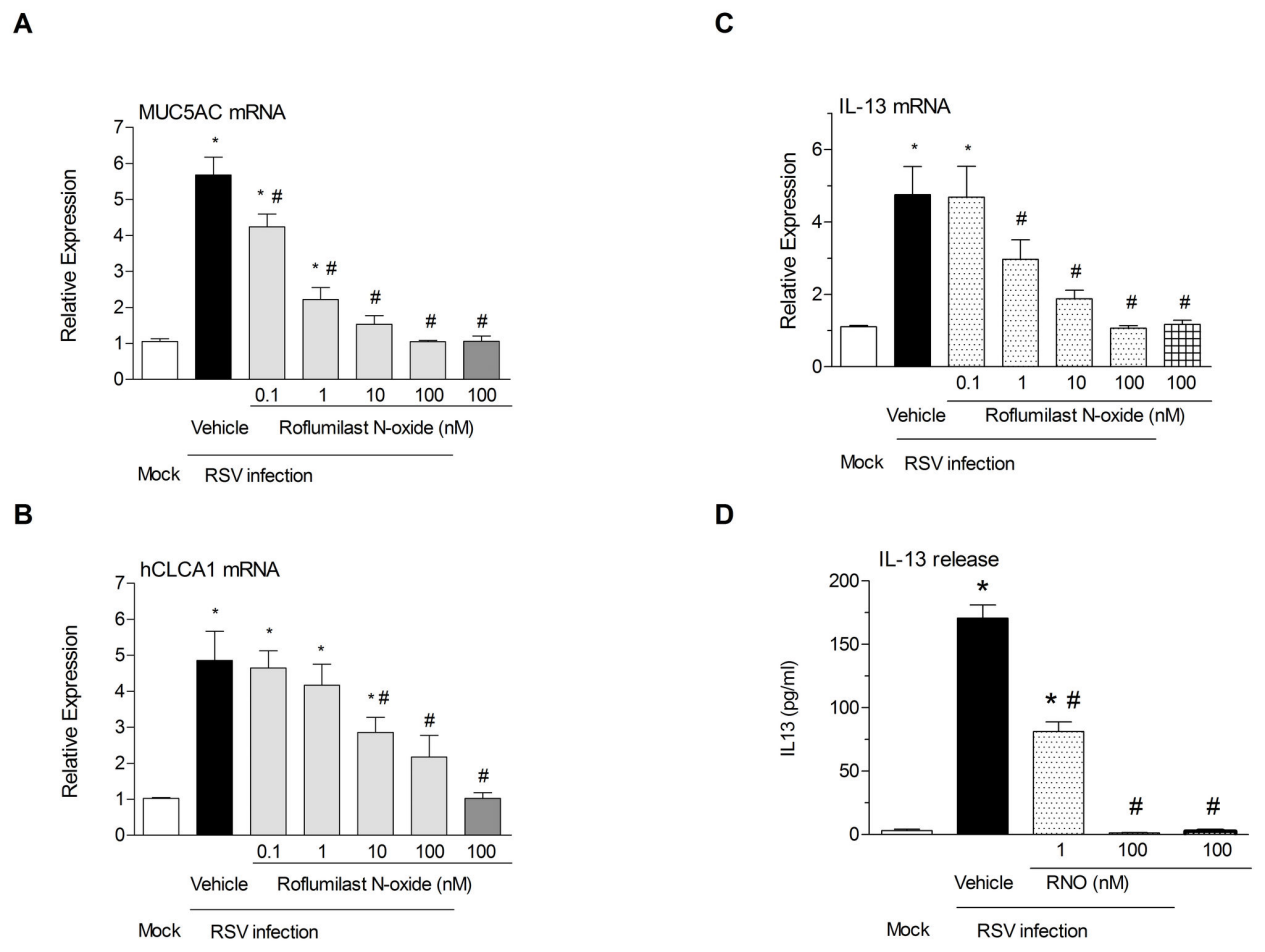
Roflumilast *N-*oxide mitigates enhanced MUC5AC and hCLCA1 mRNA expression and the expression and release of IL-13 following infection with RSV. WD-HBE were infected with RSV at 2 x 10^6^ PFU/insert in the presence of roflumilast N-oxide (0.1-100nM) or vehicle (0.1% DMSO) or mock infected and cultured until day 10 when measurements were performed. Total RNA was extracted and analyzed by real time RT-PCR for MUC5AC (panel A), hCLCA1 (panel B) and IL-13 (panel C) in mock (white bars) or RSV infected cultures in the absence (black bars) or presence (stippled bars) of roflumilast N-oxide (RNO). IL-13 release was evaluated by luminex in culture supernatants sampled as described in the Methods section. Data represent the means ± SEM of three independent infections. Each condition was evaluated by triplicate in three independent wells. Cultures from three different patients were used. * p< 0.05 compared to mock-infected cells. # p<0.05 compared to RSV infected cells.

RSV infection resulted in an increase of goblet cells and in a reduction of the thickness of the epithelial layer (verified by PAS staining, data not shown) and in an increase of MUC5AC and hCLCA1 expression. RNO prevented these alterations (Figure 3 panels A and B). EC50 values for MUC5AC and CLAC1 expression were of 0.25 and 3.8 nM respectively. As IL13 is implicated in these alterations, mRNA and protein levels were measured. RSV increased both expression and protein levels of IL13 and RNO restored them in a dose dependent manner (Figure 3 panels C and D).

### Effects of roflumilast *N-*oxide on the expression and release of IL-8, IL-6 and TNF-α from RSV infected, differentiated WD-HBE

**Figure 4:**
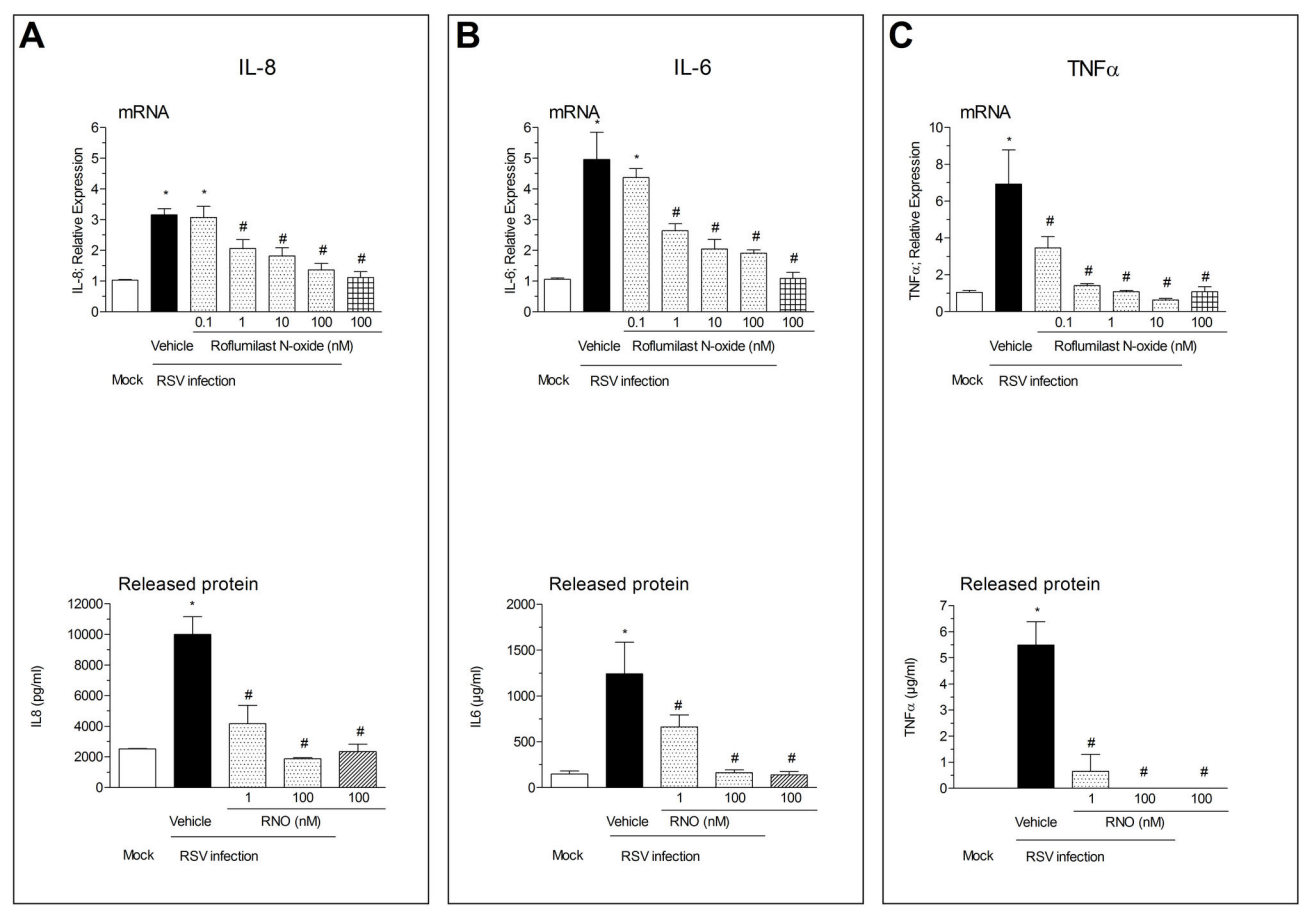
RSV infection stimulated the expression and release of IL-8, IL-6 and TNFα from WD-HBE: Prevention by Roflumilast N-oxide. WD-HBE were mock-infected or infected with RSV at 2 x 10^6^ PFU/insert in the presence of Roflumilast N-oxide (RNO; 0.1-100nM) or vehicle (0.1% DMSO). Measurements of IL-8 (panel A), IL-6 (panel B) and TNFα (panel C) mRNA transcripts (upper panels) and released protein (lower panels) were performed at day 10 after infection. For quantitative mRNA expression analyses total RNA was extracted, reverse transcribed and analyzed by real time RT-PCR. For measurements of released cytokines apical supernatants from an one hour incubation as described in Methods were collected and analyzed using Luminex. Results are depicted as the means ± SEM of three independent infections. Each condition was evaluated by triplicate in three independent wells. Cultures from three different patients were used. * p< 0.05 compared to mock-infected cells. # p<0.05 compared to RSV infected cells.

After RSV infection, we have found an increase of mRNA and protein release of IL-8, IL-6 and TNF-α in WD-HBE. This increase was inhibited by RNO concentration-dependently (EC50 of 0.6 (IL8), 0.4 (IL6) and 0.07 (TNF-α) nM for mRNA expression). Results are summarized in Figure 4.

### Effect of RNO on the compromised anti-oxidant machinery and enhanced oxidative stress in RSV-infected WD-HBE

**Figure 5:**
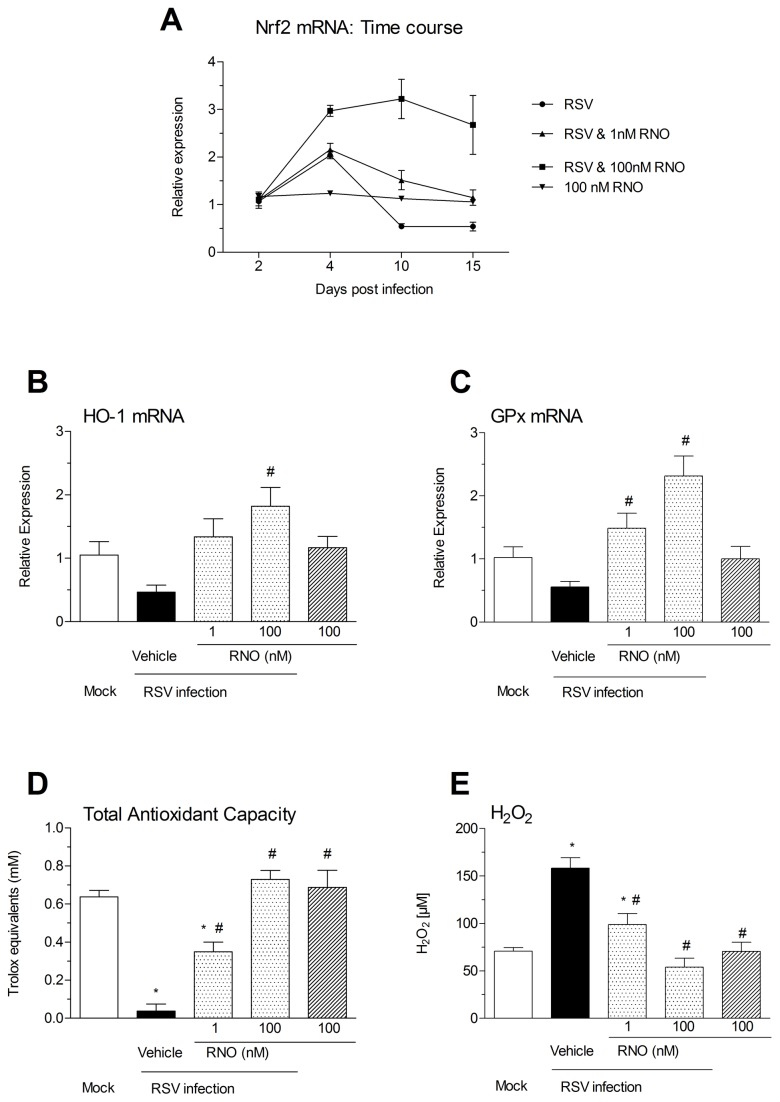
Roflumilast N-oxide supported the anti-oxidative apparatus compromised in RSV-infected WD-HBE and reduced ROS. WD-HBE were mock-infected or infected with RSV at 2 x 10^6^ PFU/insert in the presence of Roflumilast N-oxide (RNO) at 1 nM or 100 nM or vehicle (0.1% DMSO). In order to quantify Nrf2 mRNA transcripts total RNA was extracted from RSV or mock infected cultures at days 2, 4, 10 and 15 after infection, reverse transcribed and analyzed by Real Time RT-PCR (panel A). HO1 (panel B) and GPX (panel C) mRNA were analyzed with the identical procedure at day 10 after infection. Again at day 10 after RSV or mock infection Total Antioxidant Capacity (TAC) (panel D) and H_2_O_2_ release (panel E) were analyzed in cell lysates using ABTS® or Amplex Red® reagents, respectively as indicated in the Methods. Results are shown as the means ± SEM of three independent infections. Each condition was evaluated by triplicate in three independent wells. Cultures from three different patients were used. * p<0.05 compared to mock-infected cells. # p<0.05 compared to infected cells.

An imbalance between enhanced production of reactive oxygen species (ROS) and suppressed antioxidant enzymes and Nrf2 plays a critical role in RSV infection [9,11]. In the current experiments, RNO at 1nM prevented the reduced expression of Nrf2 at days 10 and 15 following RSV infection. At a concentration of 100 nM the PDE4 inhibitor further enhanced the increase in Nrf2 following RSV infection at day 4 and augmented Nrf2 by about 2.5-3 fold over mock –infected cells at all measured time points post infection (Figure 5 panel A). RSV infection of WD-HBE reduced the transcripts of HO-1 and GPX by about 50% compared with mock infected cells. RNO 1nM prevented this reduction while at 100nM increased HO-1 and GPx mRNAs by 1.8-fold and 2.2-fold over mock infected cells respectively (Figure 5 B and C). Finally, a more than 90% suppression of the total antioxidant capacity (TAC) observed at day 10 following RSV infection was reversed by about 52% by RNO at 1 nM and fully restored at 100nM (Figure 5 panel D). Further, RNO diminished the increase in intracellular H_2_O_2_ found at day 10 after RSV infection (Figure 5 panel E).

## Discussion

A key observation from the current study has been that RNO inhibits RSV infection in WD-HBE cultures which reproduce many aspects of normal human epithelium. RNO mitigates the decrease in ciliary activity as a reflect of the loss in ciliated cells, goblet cell metaplasia/hyperplasia and the increase in IL-13 and MUC5AC. RNO also inhibits the transcription and release of proinflammatory cytokines and ICAM-1 that follows RSV infection and also attenuates ROS by restoring the imbalance between its formation and a suppressed anti-oxidative capacity. Although our work has made use of a reference strain of RSV it would be interesting to carry out similar studies with a set of well characterized clinical isolates, even though Villenave et al. have reported qualitative similar effects (despite some quantitative differences) of the reference strain A2 and the clinical isolate BT2a in equivalent cultures of differentiated bronchial epithelial cells [8].

To our knowledge this is the first study that uses WD-HBE cultures generated from human lung tissue in an air-liquid interface protocol to study the protective effects of RNO against RSV infection. These cultures comprise ciliated cells, mucus producing cells and basal cells. Effects from RSV infection on WD-HBE *in vitro* as confirmed in the current work are reminiscent of the pathology of RSV-induced bronchiolitis in humans. (i) RSV target ciliated cells at their apical surface (ii). RSV infection is followed by a loss in ciliated cells and triggers goblet cell hyperplasia / metaplasia associated with an increase in MUC5AC (iii). RSV results in increased cytokines and chemokines supposed to orchestrate inflammatory cell influx characteristic of severe RSV bronchiolitis [8].

Although we haven’t measured cAMP levels directly, the EC50 values used in this study support that in this *in vitro* model of RSV infected WD-HBE cultures RNO acts by inhibiting PDE4 with consequent increase in cAMP levels [37]. This increase in cAMP levels has been previously reported in cultured WD-HBE cells, alveolar type II cells, macrophages, smooth muscle cells, epithelial intestinal cells and cardiomyocytes cells using the same range of doses studied in this work [21,43–47]. Concerning mechanisms involving in the reduction of RSV infection, speculative conclusions can only be established. RNO might interfere with mechanisms of RSV binding and entry to the (ciliated) bronchial epithelial cells. Earlier work points to ICAM-1, heparin, annexin II or TLR4 as potential “RSV receptors” on the host cell as they were found to interact with viral F (ICAM-1, TLR4) and G (heparin, annexin II) proteins [48]. Moreover ICAM-1 expression is controlled by ROS mediated events [49]. Data presented here indicate that after RSV infection there is a significant increase in the ICAM-1 mRNA levels in WD-HBE cultures which is inhibited by RNO. This inhibition could explain, at least in part, some of the anti-infective properties of RNO found and could be a direct consequence of the oxidative stress modulation observed in RNO treated cultures.

Augmented ROS in RSV infection is attributed to an imbalance between (enhanced) formation by NADPH oxidases [50] and (suppressed) degradation by the anti-oxidative enzymatic apparatus governed by Nrf2 [11]. In the current and previous work from this laboratory [9] an initial increase in Nrf2 mRNA at about 4 days post RSV infection was followed by a later loss to about half the expression level in mock infected cells at ≥ 10 d. This kinetics has also been observed in A549 cells and small airway epithelial cells [51]. Diminished antioxidant enzymes following RSV infection as found for GPx in the current study have been described *in vitro*, *in vivo* and in human [51,52]. In RSV-infected mice reduced lung activities of SOD, catalase, GPx, GST are paralleled by a loss in nuclear Nrf2. In infants with severe RSV bronchiolitis anti-oxidant enzymes are compromised. RNO, in a concentration-dependent manner, rescued from the loss in HO-1 and GPx expression following RSV indicating a potential to support the anti-oxidative apparatus. These effects likely emanate from the ability of the PDE4 inhibitor to enhance Nrf2. RNO was not only reversing the loss in Nrf2 caused by RSV but increased Nrf2 transcripts by about 3 fold over (mock-infected) controls. Likely, this increase in Nrf2 over baseline cannot only be explained by reduced viral load. A direct effect of cAMP to enhance Nrf2 as described in human keratinocytes or melanocytes is not excluded [53]. Given that sulforaphane curbs viral load in RSV infected mice [54] the enhanced Nrf2 by RNO may support to alleviate RSV infection. RNO reduced the increased generation of H_2_O_2_ in RSV-infected WD-HBE cultures. Aside from an inhibition of NADPH oxidases well described for PDE4 inhibitors [21] the enhanced GPx expression may contribute to mitigate H_2_O_2_ release by roflumilast N-oxide [51]. Taming ROS may restore Nrf2 and the anti-oxidative armamentarium [9]. Collectively, the current data suggest that RNO may correct the imbalance between enhanced ROS generation and a suppressed anti-oxidative apparatus in RSV infection. These effects can explain the inhibitory at cilia activity, metaplasia, mucin production and inflammatory cytokine and chemokines expression and release reported here, and are in parallel with those previously described for the antioxidant N-acetyl-cysteine (NAC) [9].

RSV may cause a broad spectrum of (lower) respiratory diseases that includes life-threatening bronchiolitis and respiratory failure specifically in infants and young children. RSV is among the players in acute exacerbations of COPD and asthma [55]. Roflumilast, the most advanced of PDE4 inhibitors, has been recently approved by the European Medicines Agency (EMA) for the maintenance treatment of severe COPD disease (FEV1 post-bronchodilator less than 50% predicted) associated with chronic bronchitis in adult patients with a history of frequent exacerbations as add on to bronchodilator treatment. Data presented here indicate that pre-treatment of WD-HBE cultures with RNO, the active metabolite of roflumilast, inhibits RSV infection, which is one of the viruses related to exacerbations. Roflumilast, compared to other anti-inflammatory drugs, has shown effectiveness in therapeutic models of inflammation [56]. Although in principle roflumilast is not an antiviral drug, it would be interesting to explore if this compound could have any effect in a therapeutic scenario where the viral infection was well established in the airway epithelia. More studies are necessary to answer this question. It remains an open question, whether one or another from the growing list of different therapeutic drugs that have shown to reduce viral infection of human bronchial epithelial cells and their consequences *in vitro*, to which PDE4 inhibitors are now added, may translate into a favorable outcome in controlled clinical trials.
